# Parkinson’s Disease With Psychosis: A Case Report of Neuropsychiatric Manifestations

**DOI:** 10.1192/bjo.2025.10758

**Published:** 2025-06-20

**Authors:** Aung Sein, Mobolape Olajide, Rachel Rajaratnam, Lior Mevorach, Sakshi Alewad

**Affiliations:** Essex Partnership University NHS Trust, Colchester, United Kingdom

## Abstract

**Aims:** Parkinson’s disease (PD) is mainly a movement disorder, although 30% of PD patients may also suffer from psychosis, which may impair quality of life. PD psychosis (PDPsy) may result following approximately 10 years of treatment using dopaminergic agonists. PDPsy is characterized by recurrent and continuous hallucinations and delusions lasting for at least 1 month.

**Methods:** A 59-year-old female was admitted under Section 2 of the Mental Health Act (MHA) due to concerns raised by the police and her family regarding her mental health. The police were particularly alarmed after she repeatedly contacted them, expressing paranoid delusions. Her confusion and memory issues further contributed to the concerns leading to her admission. She was under the Early Intervention Psychosis team at the time of admission but her engagement with them was very erratic. She was started on quetiapine 50 mg ON but was non-compliant. She was diagnosis with PD 12 years prior to admission, and was still under the neurology team, and being regularly reviewed. The medication prescribed for PD were Sinemet 12.5/25 2 tabs QDS and ropinirole 8 mg BD.

Upon admission, the patient reported feeling monitored via 16 satellites connected to her television, broadcasting signals worldwide. She denied calling the police and instead suggested that her phone had been hacked. The police reported that she alleged that she had been sexually assaulted by her ex-partner or individuals organized by him while being drugged, though she had no memory of these events when questioned. She believed she was being followed, a notion she first experienced on a train to Cornwall a year prior. She also alleged that her ex-partner frequently entered her home at night to steal from her. She, also, exhibited delusional beliefs regarding her YouTube presence, asserting that her ex-partner manipulated satellites to influence her views online. Risk factors identified included medication non-compliance, poor insight, risk of falls due to Parkinson’s disease, potential financial exploitation due to engagement with strangers on social media, and vulnerability.

**Results:** The differential diagnosis considered for this patient:

Delusional Disorder.

Late-Onset Psychosis.

Parkinson’s Disease Psychosis.

Alzheimer’s Disease with Psychosis.

Brief Reactive Psychosis.

A literature search showed that management of PDPsy involves a balance between reducing PD medication and introducing an antipsychotic for symptom management.

**Conclusion:** This case highlights the diagnostic challenges of psychosis in Parkinson’s disease and underscores the importance of a multidisciplinary approach in managing psychiatric symptoms in neurodegenerative conditions.

